# Gynæcology

**Published:** 1904-07-16

**Authors:** 


### GYNÆCOLOGY.

**The Etiology and Pathogenesis of Dysmenorrhœa.** There is still considerable difference of opinion as to the cause of painful menstruation in those cases in which no actual disease of the sexual organs can be found. Schultz,[1] writing on this subject, considers that the pain is due to certain changes in the musculature of the uterus, particularly in the outer layers of the uterine wall. As the full development of the muscular layers of the uterus is not reached until the age of 20 years, it sometimes happens that at the onset of menstruation the connective tissue, especially in the outer layers, is more in evidence than the muscular fibres. If complete failure of development occurs and the uterus remains infantile, amenorrhœa is the result; if development is incomplete disturbances of menstruation occur. The author considers that the curative action of parturition is due to the complete and lasting development of the muscular layers which occurs during pregnancy. Gottschalk,[2] writing on the etiology of membranous dysmenorrhœa, rejects the various theories which have ascribed it to some inflammatory condition. He has found wide-spread venous thrombosis in the vessels of the expelled mucous membrane, and thinks it probable that its separation is effected by a process of dissecting hæmorrhage, somewhat analogous to that causing premature expulsion of the placenta. Cardiac weakness with sluggish circulation is sometimes at the bottom of the matter, and a case under his care was completely cured by treatment directed to the heart, after the usual gynæcological methods had failed.

**Ventrifixation of the Uterus.**—W. J. Sinclair[3] considers that of the many operations that have been practised for chronic retroflexion that of ventrifixation is the best. He insists that in performing this operation certain principles must be kept in mind and complied with in every case. One of these is that the fundus uteri and the round ligaments must be strictly safeguarded, so that if pregnancy occurs it will run a perfectly normal course; another important point is to effect complete and permanent adhesion of a sufficient area of the anterior surface of the uterus to the abdominal wall. He has discarded all suture material except catgut and the finest silk. He obtains a broad firm adhesion between the uterus and anterior abdominal wall by means of two sutures on each side passing through the anterior surface of the uterus near its edge, and others which pass transversely through its anterior wall, the highest of these being placed about half-way between the fundus and isthmus, thus leaving the fundus free. The abdominal wound is closed in the usual way, and it is an advantage to suture separately the edges of the fascia with catgut. The last step in every case is the introduction of a glycerine pad pessary into the vagina to press forward and support the uterus during the formation of adhesions. The patient is kept in bed for six weeks. From an experience of 100 cases his results have been most satisfactory.

**Abscess in the Wall of the Uterus.**—Arnold W. Lea[4] reports an interesting case of abscess of the uterine wall in a patient who was suffering from gonorrhœa at the time of delivery. During the second week after delivery the patient had several attacks of pain in the hypogastrium; at the end of six weeks she was suddenly seized with intense abdominal pain, followed by symptoms of acute peritonitis. Abdominal section was performed and an abscess in the posterior wall of the fundus uteri was found to have ruptured into the peritoneal cavity. The abdomen was irrigated and abdomino-vaginal drainage established by means of a large drainage tube. The patient, although very ill for a few days, made a good recovery. A somewhat similar case is reported by Lepage.[5] The patient four days after delivery had a rise of temperature accompanied by a rapid pulse, vomiting and pain in the right iliac fossa. Appendicitis was diagnosed and medical treatment proved satisfactory for a few days. A fortnight later the symptoms recurred with all the signs of peritonitis. Abdominal section was performed, and an abscess which had opened spontaneously, was found in the right cornu of the uterus. The patient died on the following day.

**Operation for Repair of the Pelvic Floor.**—J. G. Mumford[6] points out that the levator ani muscle is the most important structure closing the pelvic outlet, and the one that is most frequently damaged in cases of prolapse of the uterus. He describes an operation which aims at restoring the continuity of this muscle. He first splits the recto-vaginal septum, and separates the rectum from the vagina as far up as the posterior cul de sac, when the edges of the levator ani muscle can be defined. Buried catgut sutures are now inserted, the upper three stitches pass through the fibres of the levator ani, the lower ones grasp the superficial muscles, while the last stitch of all takes up the fibres of the sphincter ani. When these deep stitches are tied a very strong and broad perineal floor is formed. A second row of buried sutures is then passed, and finally the skin wound is drawn together. The results of the operation in 40 cases were most satisfactory. Abuladse[7] recommends that a ruptured perineum should be carefully repaired during the later stage of the puerperium, the operation being performed as soon as the lochial discharge has ceased. He says that the operation when undertaken at this time is usually successful, and a stronger perineum is formed than when the tear is sutured immediately after labour. This view is quite contrary to the generally accepted opinion, but Abuladse reports a number of successful cases of his own in support of this practice.

**Inversion of the Uterus.**—Frisson[8] advocates the following treatment for inversion of the uterus:—(1) Manual reduction should be attempted in every case, whether acute or chronic. (2) Bi-lateral incision of the cervix is applicable to all acute cases and to those chronic ones in which the obstacle to reduction is demonstrated to be a constricting ring. (3) In others colpohysterotomy is the operation of election. (4) Vaginal hysterectomy is indicated in cases associated with intractable hæmorrhage or when the irreducible uterus has been so injured that there is no probability of its regaining its functional integrity. He considers hemisection the most rapid and convenient method of extirpating the organ.

**Sterility.**—Peter Horrocks,[9] in a paper on this subject, points out that for the production of a child the following conditions must be fulfilled:—(1) The ovum must be living (he thinks that in some cases of sterility the ovum dies soon after its escape from the Graafian follicle). (2) One or more living spermatozœa must be present. (3) The ovum must be impregnated by its union with a spermatozoon. (4) This union should take place inside the uterine cavity or in the uterine end of the Fallopian tube. (5) The ovum, after impregnation, must anchor inside the uterine cavity. (6) The mucous membrane lining the uterine cavity must be healthy. (7) There must be nothing to prevent the uterus developing to term, or at all events to such a time that the child is visible. With regard to treatment Dr. Horrocks thinks that food and exercise exert a considerable influence on sterility, and he recommends a plain diet with a moderate amount of physical exercise. Alcohol, as a rule, is contraindicated. If the patient's general health is bad her condition should be improved by suitable tonics. In all cases a careful examination should be made and suitable local treatment adopted.

**Tubal Pregnancy.**—An instructive case of ruptured tubal pregnancy is recorded by William Duncan.[10] The rupture occurred 19 days after conception and 10 days after the uterus had been curetted. The gravid tube was removed, and on microscopic examination it was seen that its lumen was intact and was separated from the gestation sac by a well-marked muscular layer. The gestation sac was situated in the outer part of the tube-wall; it contained well-marked chorionic villi, with a distinct epithelium consisting of the two layers known as Langhans' and syncytial respectively. There was an absence of any structure which could be described as "decidual" and Dr. Victor Bonney, who examined the specimen, thinks it probable that decidual cells are strictly the derivative of the stroma cells of the endometrium. To this absence of decidual cells is probably to be ascribed the rapidity with which a tubal gestation erodes the walls of the gestation sac and brings about early rupture. The specimen supports the view put forward by modern German authorities, that in all cases the implantation of the tubal gestation is in the muscular wall of the tube, and not (as was formerly supposed) on the surface of the tubal epithelium, and therefore within the tubal lumen. They maintain that the embryo burrows through the epithelial lining of the tube into the muscular coat and there develops.

[1] The Journal of Obstetrics and Gynæcology of the British Empire, Feb. 1904. [2] Med. Rec., Dec. 19, 1903. [3] Brit. Med. Jour., Mar. 26, 1904. [4] Lancet, Jan 23, 1904. [5] Brit. Med. Jour., Mar. 12, 1904. [6] Amer. Jour. of Med. Sci., Oct. 1903. [7] Brit. Med. Jour., Feb. 13, 1904. [8] Amer. Jour. of Med. Sci., Jan. 1904. [9] Lancet, Jan. 9, 1904. [10] Lancet, Feb. 27, 1904.

